# Estimation of Saliva Cotinine Cut-Off Points for Active and Passive Smoking during Pregnancy—Polish Mother and Child Cohort (REPRO_PL)

**DOI:** 10.3390/ijerph13121216

**Published:** 2016-12-08

**Authors:** Kinga Polanska, Anna Krol, Pawel Kaluzny, Danuta Ligocka, Karolina Mikolajewska, Seif Shaheen, Robert Walton, Wojciech Hanke

**Affiliations:** 1 Department of Environmental Epidemiology, Nofer Institute of Occupational Medicine, 91-348 Lodz, Poland; anna.krol@imp.lodz.pl (A.K.); pawel.kaluzny@imp.lodz.pl (P.K.); wojciech.hanke@imp.lodz.pl (W.H.); 2 Bureau of Quality Assurance, Nofer Institute of Occupational Medicine, 91-348 Lodz, Poland; danuta.ligocka@imp.lodz.pl; 3 Department of Biological and Environmental Monitoring, Nofer Institute of Occupational Medicine, 91-348 Lodz, Poland; karolina.mikolajewska@imp.lodz.pl; 4 Centre for Primary Care and Public Health, Blizard Institute, Barts and the London School of Medicine and Dentistry, Queen Mary University of London, E1 2AB London, UK; s.shaheen@qmul.ac.uk (S.S.); r.walton@qmul.ac.uk (R.W.)

**Keywords:** maternal cigarette smoking, environmental tobacco smoke, pregnancy, saliva cotinine, cut-off values

## Abstract

A reliable assessment of smoking status has significant public health implications and is essential for research purposes. The aim of this study was to determine optimal saliva cotinine cut-off values for smoking during pregnancy. The analyses were based on data from 1771 women from the Polish Mother and Child Cohort. Saliva cotinine concentrations were assessed by high performance liquid chromatography coupled with tandem mass spectrometry (HPLC-ESI + MS/MS). The saliva cotinine cut-off value for active smoking was established at 10 ng/mL (sensitivity 96%, specificity 95%) and for passive smoking at 1.5 ng/mL (sensitivity 63%, specificity 71%). About 5% of the self-reported non-smoking women were classified as smokers based on the cotinine cut-off value. Significantly more younger, single, and less educated self-reported non-smokers had a cotinine concentration higher than 10 ng/mL compared to those who were older, married, and who had a university degree. Close to 30% of the non-smokers who indicated that smoking was not allowed in their home could be classified as exposed to passive smoking based on the cut-off value. The study suggests that self-reported smoking status is a valid measure of active smoking, whereas in the case of passive smoking, a combination of questionnaire data and biomarker verification may be required.

## 1. Introduction

Due to public health and regulatory activities during the last 20 years the prevalence of active and passive maternal smoking during pregnancy has declined; however, it is still an important risk factor for adverse pregnancy outcomes, children’s health and neurodevelopment [1]. A reliable assessment of smoking status in pregnancy has significant public health implications and is essential for research purposes. Studies have shown that self-report of tobacco use and environmental tobacco smoke (ETS) exposure can be inaccurate due to the lack of acceptability, embarrassment and denial or may result from poor recall [2,3]. In addition, missing data in medical records on self-reported smoking seems to be a common problem [4]. This indicates the need for reliable assessment of exposure using standardized methods such as questionnaires and biomarkers.

Cotinine, the major metabolite of nicotine, is a frequently used biomarker of active and passive smoking [2,3,5,6,7,8]. A major advantage of using cotinine rather than nicotine as a biomarker of smoking status is that about 72% of nicotine is converted to cotinine, and the half-life of cotinine averages about 17 h in comparison to 2–3 h in the case of nicotine [2,6,9,10]. Cotinine can be measured in various biological specimens, including plasma, saliva, urine and hair [2,6].

Previous studies have recommended saliva cotinine cut-off concentrations ranging from 1 ng/mL to 24 ng/mL to indicate active smoking among pregnant women [2,9,11,12,13,14,15]. It is important to be aware of interindividual variability in metabolism of nicotine and cotinine [16], which may be partly under genetic control [17]. Studies have indicated differences in biomarker concentrations according to gender and ethnicity [2]. In addition, like many other physiological processes, metabolism of nicotine changes during pregnancy, and optimal cut-off points stated for the general population are likely to differ from those determined in pregnant women [2]. The observed variability in the metabolic clearance of cotinine may markedly increase during pregnancy, resulting in shortening of cotinine half-life by nearly 50% in comparison with the non-pregnant state [18,19]. The optimal cut-off for the classification of smoking status can also vary depending on patterns of active and passive smoking as well as on the limit of detection and quantification of methods used for biomarker assessment. In this respect, cut-off values stated previously might not be suitable for current exposure levels.

Determination of a cut-off value for passive smoking is even more challenging. The concentration of a biomarker in a passive smoking assessment is related to many factors, including exposure time, room size, ventilation and number of people smoking. Moreover, patterns and degree of exposure in different environments of daily living are likely to vary widely [7,20,21,22,23]. Taking into account policy regulations and increasing public awareness, in recent years exposure to ETS has significantly decreased in the developed countries. In such a situation, asking traditional questions about husband smoking or exposure to passive smoking at home, at work and/or in public places might not indicate exposure level accurately.

The cut-off values for distinguishing active smokers from nonsmokers, and those who are passively exposed versus non-exposed amongst non-smokers in pregnant women are yet to be clearly defined. The aim of this study was to establish optimal saliva cotinine cut-off values for active and passive smoking during pregnancy and to validate self-reported smoking status using cotinine concentration as a biomarker.

## 2. Materials and Methods

### 2.1. Study Design and Population

The analyses are based on data from the Polish Mother and Child Cohort (REPRO_PL cohort)—a multicenter prospective cohort established in 2007 with the aim of evaluating environmental and lifestyle-related factors contributing to pregnancy outcomes, children’s health and neurodevelopment. A detailed description of the cohort methodology has been published elsewhere [24]. Briefly, the women were recruited into the study if they fulfilled the following criteria: single pregnancy up to the 12th week of gestation, no assisted conception, no pregnancy complications and no chronic diseases as specified in the study protocol. The study subjects were interviewed once in each trimester of pregnancy to collect and update information about environmental, occupational and lifestyle factors, sociodemographic data, medical, and reproductive history. In addition, during each visit and after the delivery, biological samples (including saliva, urine, blood, cord blood, and hair) were collected.

The current analysis was performed based on data from 1771 women. In the course of the study, some missing data were noted including: (1) data related to the questions dedicated to smoking status assessment; (2) specific socio-demographic data (age, marital status, parity, educational level); (3) data on cotinine levels in different periods of pregnancy (a consequence of the lack or not enough saliva samples, lack of financial resources for the cotinine assessment or usage of the samples for other purposes); (4) data on the follow-up visits resulting from miscarriage, refusal to participate or changes of the place of residence.

During the first clinical visit all women in the study were informed by a gynecologist/midwife about healthy lifestyle during pregnancy (including a recommended diet, physical activity, elimination of some exposures in the workplace, alcohol consumption and smoking).

The study was approved by the Ethical Committee of the Nofer Institute of Occupational Medicine (NIOM), Łódź, Poland (ID number 7/2007), and written consent was obtained from all the study subjects.

### 2.2. Questionnaire Data

Detailed questionnaire data were obtained from all the women participating in the study (at 8–12, 20–24 and 30–34 weeks of pregnancy). Smoking status during the first trimester of pregnancy was assessed based on the following question: “Have you ever smoked cigarettes?”. Women who answered “no” were considered to be non-smokers. Those who answered “yes” were asked to answer an additional question “Have you smoked in the current pregnancy?”(if “no”, they were defined as non-smokers; if “yes”, they were asked additional questions regarding duration and number of cigarettes smoked). Women who reported smoking even a puff in the past seven days preceding the interview were considered to be smokers. The smoking status was updated during the second and third trimesters of pregnancy by asking the same questions evaluating the current smoking status (“Do you currently smoke cigarettes?” if “yes” the women were asked about the number of cigarettes smoked per day, if “no” they were asked about the week of pregnancy when they stopped smoking). The questions were asked to all the women participating in the study independently of their declaration stated in the proceeding questionnaire.

Passive smoking was assessed using three separate questions: (1) “Does your husband/partner smoke cigarettes?” (“husband/partner smoking”); (2) “Have you been exposed to passive smoking?” (“ETS”) and (3) “Is smoking allowed at your home?” (“smoking allowed at home”). This information was updated by asking the same questions during the second and third trimester interviews.

### 2.3. Saliva Collection and a Cotinine Concentration Assessment

The detailed description of the sample collection and analysis has been published elsewhere [9]. Briefly, at enrolment and each follow-up visit the women provided saliva sample into a Salivette with citric acid (Sarstedt, Nümbrecht, Germany). The amount of approximately 1–2 mL of saliva was easily obtained by having the women chew a cotton swab, at least 30 min after eating or drinking. A clear, fluid sample obtained by centrifuging the Salivette was used for the analysis. The cotinine concentration in saliva was analyzed at NIOM using the high performance liquid chromatography coupled with tandem mass spectrometry/positive electrospray ionisation (HPLC-ESI + MS/MS) and an isotope dilution method. This procedure has been validated under ISO 17025 criteria and accredited by the Polish Center of Accreditation (Certificate AB215). The limit of detection (LOD) was 0.12 ng/mL and the limit of quantification (LOQ) was 0.4 ng/mL.

### 2.4. Covariates

The following covariates, obtained by the questionnaire, were included in the analysis: maternal age at delivery (<30, ≥30 years), marital status (formally married, single), parity (0, ≥1 previous deliveries) and maternal education (highest level of completed education: ≤9, 10–12, >12 years).

### 2.5. Statistical Analysis

The data set used for the analysis is provided in the Supplementary Materials section (Table S1). In the first part of the analysis, the optimal cotinine cut-offs for detecting exposure to tobacco smoke were determined via the ROC (Receiver Operating Characteristic) curve. The self-reported exposure to tobacco smoke (yes/no, separately for active and passive smoking) was regarded as the “true” indicator of the exposure, and cotinine concentration was the numeric “test” variable (for which an optimal cut-off value was determined). In this setting, sensitivity of the test was the proportion of subjects who had saliva cotinine concentration higher than the specified cut-off (hence were identified as smokers, active or passive, by the cotinine value) among the self-reported smokers (active or passive). Similarly, specificity refers to the proportion of pregnant women who had a saliva cotinine concentration less than the specified cut-off (hence were identified as non-smokers, active or passive by the cotinine value) among the self-reported non-smokers (active or passive). The optimal cotinine cut-off was the cotinine concentration for which the sum of sensitivity and specificity attained the maximum value. The cotinine cut-offs determined this way had an optimal concordance between the cotinine criterion and the questionnaire.

Bootstrap resampling experiments were run to determine non-parametric confidence levels for the optimal cotinine cut-off value and corresponding sensitivity and specificity for this cut-off. This procedure consists of repeated threshold optimization on *n* = 2000 bootstrap datasets obtained by resampling of the original data (with replacement). Resampled data kept the original proportion between the (yes/no) cases of the “true” variable.

In the second part of the analysis, the optimal cotinine cut-offs were applied as quasi-objective dichotomous indicators of exposure and tabulated with questionnaire based smoking assessments, this time treated as the “test” variables. Accordingly, sensitivity, specificity, positive and negative predictive values were calculated. The analyses were performed for all the women and for the women grouped by age, marital status, parity and education. The confidence intervals for these parameters were obtained with the exact method for binomial proportion.

The data were available for each trimester of pregnancy; however, taking into account the fact that for about 40% of the samples collected during the second trimester of pregnancy the cotinine levels were not assessed (as a result of financial and organizational reasons), cut-off estimations were restricted to the first and third trimesters.

The public domain R program (version 3.2) was used for statistical computing and graphics [25]. The R library packages pROC (version 1.8) and OptimalCutpoints (version 1.1) obtained from the CRAN repository were used for plotting ROC curves, determination of optimal cut-offs, calculation of classification quality measures (sensitivity and specificity) and corresponding confidence intervals [26,27].

## 3. Results

### 3.1. Characteristics of the Study Population

Socio-demographic characteristics of the study population are presented in the Supplementary Materials section (Tables S1–S4). More than half of the study participants were younger than 30 years and a similar proportion of them were pregnant for the first time. A high percentage of the women were married (78%) and had a university degree (64%).

### 3.2. Self-Reported Active and Passive Smoking Status

About 12% of the women declared tobacco smoking at the time of their first interview scheduled within the study and 9% in the third trimester of pregnancy (Table S1). A higher proportion of smokers was observed among the younger, unmarried and less educated women. Of all the women, more than 30% indicated that their husbands/partners smoked cigarettes, close to half of them declared exposure to passive smoking (any place), and 23% that smoking was allowed at their home (Table S2). These percentages were lower for the same questions asked in the third trimester of pregnancy, especially in the case of declared ETS exposure, which dropped from 46% to 21% through the pregnancy period (Tables S2–S4). Similar to the pattern observed among the active smokers, passive smoking was more common among the younger, single women and those with a lower educational level (Tables S2–S4).

### 3.3. Cotinine Concentrations

Saliva cotinine concentrations according to self-reported smoking status are presented in Table 1. Among the women who declared active smoking during pregnancy, the mean cotinine concentration was higher than 75 ng/mL, whereas among the non-smokers it was about 1 ng/mL. In the group of non-smoking women who declared that their husband/partner smoked cigarettes, the mean cotinine concentration was about 2 ng/mL. Among the women categorized as passive smokers based on their declaration on ETS exposure and acceptability of smoking at home for all except ETS exposure in the first trimester, mean cotinine concentrations were higher than that noted for the husband/partner smoking (>2.8 ng/mL). In addition, significantly higher mean cotinine levels were observed for the women indicating ETS exposure in the second and third trimesters of pregnancy than for the first trimester (*p* < 0.001). The cotinine concentrations in each trimester of pregnancy were highly correlated (*p* < 0.001) (Table 2).

### 3.4. Determination of Cotinine Cut-Off

**Figure 1:**
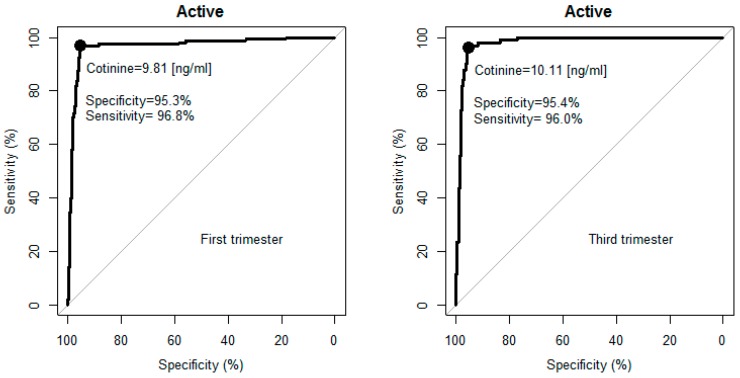
Receiver Operating Characteristic (ROC) curves for active smoking in the first and third trimesters.

The ROC analysis showed that the optimal cut-off value separating smokers from non-smokers was 9.8 ng/mL (sensitivity 97%, specificity 95%) for the first, and 10.1 ng/mL (sensitivity 96%, specificity 95%) for the third, trimester of pregnancy (Figure 1, Table 3).

**Figure 2:**
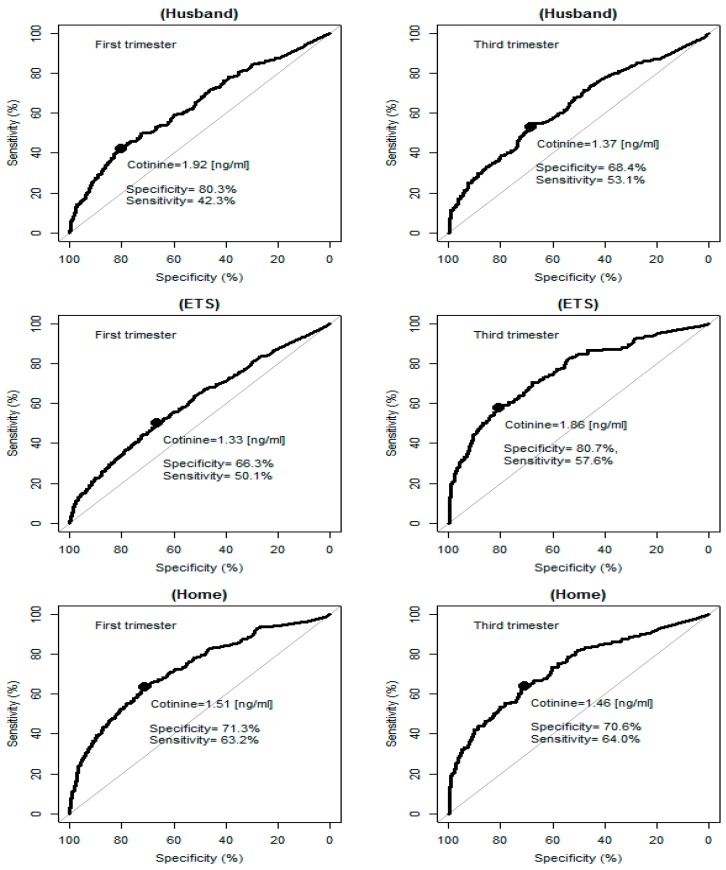
ROC curves for the first and third trimester’s passive smoking by individual sources of exposure. ETS: environmental tobacco smoke.

The series of analyses performed to estimate cut-off point for passive smoking based on different questions indicated that the values ranged from 1.3 ng/mL for ETS to 1.9 ng/mL for husband/partner smoking in the first trimester of pregnancy (Figure 2, Table 3). Taking into account sensitivity (63% for the first and 64% for the third trimester of pregnancy) and specificity (71% for the first and the third trimester of pregnancy), the optimal cut-off point was 1.5 ng/mL and the best question describing ETS was that indicating acceptance of smoking at home. Another observation from the ROC curves on Figure 2, concerning passive smoking, is that the sensitivity of the cotinine test tended to be smaller than its specificity.

The bootstrap resampling experiments confirmed the selected cut-off values (Table 3) and indicated a range of variability of the optimal cut-offs and attainable test quality, which are attributable to sampling of the subjects. For detection of active smoking status during the first and the third trimester the median cotinine cut-offs were near 10 ng/mL, and the quality of smoking detection sensitivities and specificities were also at usable levels of >90%, but the lower band of the 95% confidence interval for the median of cotinine cut-offs obtained from the third trimester data was lower (4.2 ng/mL) than for the first trimester (9.5 ng/mL). This may indicate a tendency to reduce smoking intensity by some smokers towards the end of pregnancy.

For detection of passive smoking, resampling experiments showed that the lower confidence limits for sensitivity and specificity of the cotinine test were fluctuating near the 50% chance level, which indicates that subjective, self-assessed presence or absence of passive exposure bore no resemblance to the objective assessment of exposure as attempted by the cotinine test.

### 3.5. Cotinine Cut-Off and Self-Reported Smoking and Passive Smoking Status

The saliva cotinine cut-offs (10 ng/mL for active and 1.5 ng/mL for passive smoking) were further used to validate the self-reported smoking status according to the background variables (age, marital status, education and parity) (Table 4 and Table 5). Histograms of log cotinine in each trimester of pregnancy and self-reported active and passive smoking status are presented in the Supplementary Materials section (Figure S1). About 5% of the self-reported non-smoking women were classified as smokers based on the cotinine cut-off (with the geometric mean of 55.3 ng/mL (for the first) and of 52.6 ng/mL (for the third trimester of pregnancy)). In addition, 3% of the smokers in the first trimester and 4% in the third trimester of pregnancy in cotinine assessments were recognized as non-smokers (with the geometric mean of 1.1 ng/mL (for the first) and of 3.2 ng/mL (for the third trimester of pregnancy). Thus, the percentage of smokers increased from 12% (based on questionnaire) to 15% (based on cotinine cut-off) in the first trimester, and from 9% (based on questionnaire) to 13% (based on cotinine cut-off) in the third trimester of pregnancy. Significantly more younger, single and less educated self-reported non-smokers had a cotinine concentration higher than 10 ng/mL comparing to older, married women and those with a university degree (*p* < 0.05). Close to 30% of the non-smokers who indicated that at their home smoking was not allowed could be classified as being exposed to passive smoking based on the selected cut-off (1.5 ng/mL). In addition, about 40% of the non-smokers who indicated exposure to passive smoking had a cotinine concentration below 1.5 ng/mL. The highest proportion of those who declared that smoking was not allowed at home, but had a cotinine concentration above 1.5 ng/mL, was observed among the less educated women.

The results of application of the cotinine test for detection of the smoking status in subgroups of subjects reveal noticeable differences in the agreement between objective (cotinine) and subjective (questionnaire) assessments. In the case of active smoking, specificity was stable across educational level but sensitivity had a downward trend with increasing education (Table 4). Negative predictive values increased with education, whereas positive predictive values were fairly high and remained stable across subject subgroups. Concerning passive smoking, there was noticeable increase of specificity in educated mothers (Table 5).

## 4. Discussion

This prospective cohort study suggests a saliva cotinine concentration of 10 ng/mL as the best cut-off for distinguishing active smokers from non-smokers, and 1.5 ng/mL for separating passive from non-passive smokers in the population of pregnant women. Based on our results we propose that self-reported smoking status is a valid measure of active smoking, whereas in the case of ETS exposure, a combination of questionnaire data and biomarker verification may be required.

We established a saliva cut-off value for active smoking status during pregnancy of 10 ng/mL, which is within the range previously reported in other studies (1–24 ng/mL) [2,9,11,14,15]. Based on a comprehensive review of biomarker verification of tobacco use and cessation, similar to our results, 10 ng/mL was recommended for the cut-off concentration to distinguish pregnant smokers from non-smokers [2]. What is important is that the cut-off stated for pregnant women is lower than that stated for the general population [2]. Our previous assessment based on the REPRO_PL cohort indicated a cut-off value of 12.9 ng/mL for saliva cotinine concentration [9]. That value is slightly higher than that noted in the current study, but was based on a much smaller sample size (69 pregnant women compared to 1771). Furthermore, the previously defined cut-off value is equal to the upper confidence interval in the current study. In a large prospective study of Norwegian pregnant women the cut-off point for plasma cotinine concentration was 5.3 ng/mL, which is lower than that observed in our study [4]. Much lower cut-off values for saliva cotinine were noted for Alaska native pregnant women [11]. The differences between studies may result from many factors, including the ethnicity of the population, definition and prevalence of active (occasional and current smokers) and passive smoking, as well as methods used for cotinine assessment (including its LOD and LOQ). Some race differences in cut-off values have been reported thus, generalization of cut-off based on one population might not be appropriate for other ethnic groups. As the example, in the study by Benowitz et al. the following optimal serum cotinine concentrations for discriminating smokers from non-smokers were proposed: 5.92 ng/mL, 4.85 ng/mL, and 0.84 ng/mL for non-Hispanic blacks, non-Hispanic whites, and Mexican Americans, respectively [5]. In addition, some policy and interventional activities that came into play in recent years might have led to a decrease in cut-off point over time as a result of reductions in smoking intensity or ETS exposure [11].

The sensitivity and specificity in our study were 96% and 95%, respectively, when self-reported smoking status was considered as the “true” test (gold standard). However, sensitivity dropped to about 70% when the cotinine concentration rather than self-reporting was used as the “true” test (gold standard). This has also been observed in other studies [5]. In our study, about 5% of self-reported non-smoking women were classified as smokers based on the cotinine cut-off. This can result from underreporting of smoking status or high ETS exposure [28]. Of 60 women (self-reported non-smokers but classified as smokers based on biochemical verification) 75% had a cotinine level higher than 25 ng/mL, so they can be recognized as smokers with an even higher cut-off value. We have also noted that there were 16 women who declared non-smoking status in the first trimester of pregnancy and described themselves as smokers when asked a similar question in the third trimester of pregnancy. This also proves that underreporting of smoking status cannot be excluded. Among the women who had a cotinine level between 10–25 ng/mL only one indicated no passive smoking in all three questions stated for that assessment which can confirm that higher cotinine levels can result from high ETS exposure. Significantly more younger, single and less educated self-reported non-smokers had a cotinine concentration higher than 10 ng/mL, compared to older, married women and those with a university degree. A similar pattern was observed in a study by Kvalvik et al. [4]. In addition, about 3% of self-reported smokers were recognized as non-smokers by cotinine assessments. As it is unlikely that non-smokers report themselves as smokers, it is possible that a low cotinine concentration may result from an occasional smoking. Based on the number of cigarettes smoked per day indicated by the women, about 4% of them smoked less than 1 cigarette/day. In general we would argue that self-reported smoking status is a valid measure of active smoking, but for some groups (for example those of lower educational and socioeconomic status) verification of self-reported non-smoking might be needed. It needs to be highlighted that this conclusion is true for our study population. A good standardized questionnaire is crucial for an assessment of smoking status. The part of our questionnaire dedicated to smoking assessment was developed by the team of experts in this field. The women were interviewed by trained midwives with whom they met few times in pregnancy period (so if they wanted to lie about their smoking status they needed do so also during subsequent visits). In addition, the midwives usually knew the women as they are covered by the same institution (clinic) for health care. The women were also informed about the purpose of saliva sample collection so they could suspect that the smoking status would be verified. What is also important is that our study did not only focus on tobacco smoking (the questions dedicated to smoking status assessments were incorporated into a very extensive questionnaire dedicated to a variety of factors that might have impact on pregnancy outcomes, children’s health and neurodevelopment) so this can also have the impact on a declaration regarding smoking status. In our study, the saliva cotinine cut-off point to differentiate non-smokers exposed to passive smoking from non-exposed individuals was 1.5 ng/mL. In the study by Sasaki et al. the recommended plasma cotinine cut-off in the group of pregnant women was 0.21 ng/mL, which is much lower compared to our results [20]. It should be emphasized that a cut-off point for passive smoking is less clear and more controversial than the cut-off for active smoking. Furthermore, defining a cut-off for passive smoking is particularly challenging, given the variety of sources of exposure. It seems that ETS assessment based on husband/partner’s smoking status or exposure in public places might lead to misclassification. This may be especially so during pregnancy. If we take into account the fact that people are aware of health consequences of ETS and do not accept it, pregnant women might describe their exposure inaccurately. The results of a study performed in California in 1992 suggested that a question about the number of smokers in the household can account for a statistically significant amount of variation in serum cotinine concentrations [23]. At the same time, the authors indicated that the use of this question alone does not provide an adequate estimation of exposure as compared with cotinine concentration. Smoking by family members does not necessarily equate to exposure to ETS, as the person may refrain from smoking in the presence of a pregnant woman. On the other hand, some level of exposure can be detected as the result of exposure to nicotine in the air emanating from a smoker’s clothes or hair, even when the smoker has not smoked at home [23]. Another important issue which needs to be considered when assessing ETS exposure using biomarkers is that the intake of food and drinks containing nicotine (like tomatoes, tea and coffee) might falsely indicate exposure to ETS. An interesting aspect of our study is that, among the women categorized as passive smokers based on their reporting of ETS exposure and acceptability of smoking at home, for all except ETS exposure in the first trimester, the mean cotinine concentrations were higher than those seen in relation to reporting of the husband/partner smoking. In addition, mean cotinine levels were significantly lower for ETS in the first comparing to the second and third trimesters of pregnancy. The differences noted in the case of the first trimester of pregnancy can result from an inaccurately stated question (which has not adequately addressed the time of exposure, whereas for the other trimesters the current exposure was estimated). Our results, together with other studies in this field, underline the need to consider sources of exposure other than partner’s tobacco smoke, and to assess them individually together with biochemical verification, rather than as an unweighted summative measure [22].

Strengths of the current study include the large sample size of pregnant women from the prospective Mother and Child Cohort (REPRO_PL), which provides more precise estimates, and the repeated cotinine measurements throughout pregnancy and the questionnaire data obtained at the time of sample collection. A current state-of-the-art method for determination of cotinine in biological material is liquid chromatography with mass spectrometry (LC-MS/MS) and atmospheric pressure or electrospray ionization [8]. The results of our study offered a method characterized by improved sensitivity, selectivity, and high throughput in comparison to other conventional techniques.

The study also has some limitations. Firstly, although the detailed questionnaire data were collected and updated through pregnancy, there may have been some misclassification of the exact timing of exposure (especially in the case of ETS). Secondly, as this cohort study was set up to evaluate the impact of a broad variety of factors on pregnancy outcomes and children’s health, and was not only focused on the effects of tobacco exposure, information about other forms of nicotine exposure are missing. However, based on the Global Adult Tobacco Survey (GATS) that was conducted in Poland in 2009/2010, only about 0.1% of the adult population described themselves as current smokeless tobacco users [29]. Moreover, e-cigarettes have only been available on the Polish market since early 2008 with increased popularity in recent years, so in the case of our study (conducted in 2007–2010) they are of less importance [30]. As nicotine replacement therapy (NRT) at the time of the study was not recommended during pregnancy in Poland (taking into account lack of data about its safety and effectiveness), this exposure is unlikely to have been common in our population [31,32]. In addition, taking into account its half-life, cotinine could not be recognized as a “gold standard” as it does not provide the measure of long-term exposure, which is especially crucial in the case of pregnancy. Some missing data for selected variables and subsequent visits constitute an additional limitation of the study. However, as the study was not dedicated only to smoking and its determinants we do not expect that refusals were related to the smoking status. Finally, self-selection bias cannot be excluded. The pregnant women were informed that the sample collection would be used for research purposes, hence it is possible that they might have refrained from smoking or limited their ETS exposure prior to saliva sampling (especially in the case of subsequent visits). This may have also influenced their self-reported smoking status. The prevalence of smoking in the pregnant women in our study is in agreement with data from a survey conducted in Poland which found that 25% of women smoked three months prior to conception, and that 12% continued to smoke during pregnancy [33]. The other study conducted in Poland has indicated a higher proportion of smokers among pregnant women [34]. This can be explained by socio-demographic differences between the populations (mostly related to the place of residence and educational level of the study subjects: big cities and highly educated population in our study vs. small towns with less educated women in the study by Balwicki et al.) [34].

The results of our study have important implications, not only for research purposes, but also for public health practice. In order to identify pregnancies at risk, public health practitioners need to have an objective measure of tobacco exposure [11]. Based on our data, verification of self-reported smoking status by biochemical measurements might be needed in the case of less educated women and for appropriate ETS assessment. Saliva cotinine measurements are an easy, non-invasive way to assess tobacco exposure status and to obtain data which could be used to inform public health intervention efforts [11].

## 5. Conclusions

In conclusion, a saliva cotinine cut-off value of 10 ng/mL was established for distinguishing pregnant active smokers from non-smokers, and a cut-off of 1.5 ng/mL for distinguishing exposed from unexposed nonsmokers. The current study suggests that self-reported smoking status is a valid measure of active smoking, whereas in the case of environmental tobacco smoke exposure, a combination of questionnaire data and biomarker verification may be required in pregnant women.

## Figures and Tables

**Table 1:** Saliva cotinine concentrations according to self-reported active and passive smoking status during pregnancy.

Tobacco Smoke Exposure *	*n*	Geometric Mean (ng/mL)	95% CI (ng/mL)
Non-smokers			
1st trimester (*n* = 1505; missing = 231)	1274	1.14	1.06–1.22
2nd trimester (*n* = 1356; missing = 554)	802	1.10	1.01–2.00
3rd trimester (*n* = 1273; missing = 293)	980	1.15	1.07–1.24
Smokers			
1st trimester (*n* = 196; missing = 39)	157	78.77	65.53–94.68
2nd trimester (*n* = 142; missing = 36)	106	98.48	81.83–118.52
3rd trimester (*n* = 122; missing = 23)	99	85.84	69.75–105.65
Passive smokers **			
1st trimester husband/partner smoking (*n* = 423; missing = 61)	362	1.84	1.56–2.16
2nd trimester husband/partner smoking (*n* = 338; missing = 148)	190	2.08	1.66–2.60
3rd trimester husband/partner smoking (*n* = 325; missing = 82)	243	1.89	1.55–2.31
1st trimester ETS exposure (*n* = 614; missing = 99)	515	1.55	1.36–1.76
2nd trimester ETS exposure (*n* = 236; missing = 106)	130	3.24 ^a^	2.34–4.49
3rd trimester ETS exposure (*n* = 204; missing = 65)	139	3.42 ^b^	2.57–4.56
1st trimester smoking allowed at home (*n* = 239; missing = 30)	209	2.86	2.27–3.61
2nd trimester smoking allowed at home (*n* = 185; missing = 80)	105	3.53	2.51–4.96
3rd trimester smoking allowed at home (*n* = 181; missing = 45)	136	3.19	2.37–4.28

^a^ Environmental tobacco smoke (ETS) 1st trimester vs. ETS 2nd trimester, *p* < 0.001 (nonparametric Wilcoxon test); ^b^ ETS 1st trimester vs. ETS 3rd trimester, *p* < 0.001 (nonparametric Wilcoxon test); * the numbers might not sum up to the total sample size as some missing data were observed (described in the Materials and Methods section of the manuscript); ** restricted to non-smokers; CI: confidence interval.

**Table 2:** Correlations between cotinine concentrations in the first, second and third trimesters of pregnancy.

Cotinine Concentrations *	1st Trimester	2nd Trimester	3rd Trimester
1st trimester	(1464)	0.85	0.62
2nd trimester	(923)	(924)	0.79
3rd trimester	(1039)	(863)	(1102)

* Spearman correlation coefficients are in the upper triangle of the table. All correlation coefficients are significantly different from 0 with *p* < 0.0001. The values in parenthesis denote number of the subjects with available pairwise cotinine measurements for the respective trimesters; the numbers might not sum up to the total sample size as some missing data were observed (described in the Materials and Methods section of the manuscript).

**Table 3:** Variability of optimal cotinine cut-off points for maternal smoking status, and corresponding sensitivity and specificity of cotinine tests using the optimal cut-offs to predict reported smoking status.

Smoking Status *	Median	95% CI
Active smoking		
1st trimester: optimal cut-off	9.8	9.5–12.9
1st trimester: optimal sensitivity	96.8	93.6–99.4
1st trimester: optimal specificity	95.4	94.2–96.4
3rd trimester: optimal cut-off	10.1	4.2–13.5
3rd trimester: optimal sensitivity	97.0	92.9–100.0
3rd trimester: optimal specificity	95.3	91.6–96.7
Passive smoking		
1st trimester: optimal cut-off	1.52	1.2–2.2
1st trimester: optimal sensitivity	63.6	48.8–77.5
1st trimester: optimal specificity	72.1	58.9–84.8
3rd trimester: optimal cut-off	1.5	0.9–2.9
3rd trimester: optimal sensitivity	66.2	43.4–85.3
3rd trimester: optimal specificity	71.3	50.2–90.4

* The values were obtained using the stratified bootstrap resampling (*n* = 2000); 95% CI for the median of optimal cut-off values; sensitivity and specificity of smoke exposure status with the cotinine test based on optimal cut-offs.

**Table 4:** Sensitivity (Se), specificity (Sp), positive and negative predictive values (PV+, PV−) according to the saliva cotinine cut-off of 10 ng/mL.

Characteristics	Non-Smokers *n* (%)		Smokers *n* (%)		Se (95%CI)	Sp (95% CI)	PV+ (95% CI)	PV− (95% CI)
	<10 ng/mL	≥10 ng/mL	<10 ng/mL	≥10 ng/mL				
**the 1st trimester of pregnancy**								
All the women	1214 (95.3)	60 (4.7)	5 (3.2)	152 (96.8)	71.7 (65.1–77.7)	99.6 (99.0–99.9)	96.8 (92.7–99.0)	95.3 (94.0–96.4)
Maternal age (years)								
<30	667 (93.8)	44 (6.2) *	4 (3.7)	103 (96.3)	70.1 (62.0–77.3)	99.4 (98.5–99.8)	96.3 (90.7–99.0)	93.8 (91.8–95.5)
≥30	535 (97.1)	16 (2.9)	1 (2)	48 (98)	75 (62.6–85.0)	99.8 (99.0–100)	98 (89.1–99.9)	97.1 (95.3–98.3)
Parity								
0	638 (95.7)	29 (4.3)	2 (2.7)	73 (97.3)	71.6 (61.8–80.1)	99.7 (98.9–100)	97.3 (90.7–99.7)	95.7 (93.8–97.1)
1	407 (95.8)	18 (4.2)	2 (3.5)	55 (96.5)	75.3 (63.9–84.7)	99.5 (98.2–99.9)	96.5 (87.9–99.6)	95.8 (93.4–97.5)
≥2	169 (92.9)	13 (7.1)	1 (4)	24 (96.0)	64.9 (47.5–79.8)	99.4 (96.8–100)	96 (79.6–99.9)	92.9 (88.1–96.1)
Marital status								
Married	996 (96.8)	33 (3.2) **	3 (3.4)	84 (96.6)	71.8 (62.7–79.7)	99.7 (99.1–99.9)	96.6 (90.3–99.3)	96.8 (95.5–97.8)
Single	217 (88.9)	27 (11.1)	2 (2.9)	68 (97.1)	71.6 (61.4–80.4)	99.1 (96.7–99.9)	97.1 (90.1–99.7)	88.9 (84.3–92.6)
Maternal education								
≤9	27 (77.1)	8 (22.9) *^,a^	0 (0)	35 (100)	81.4 (66.6–91.6)	100 (81.7–100)	100 (85.5–100)	77.1 (59.9–89.6)
10–12	326 (91.1)	32 (8.9) **^,b^	3 (3.6)	80 (96.4)	71.4 (62.1–79.6)	99.1 (97.4–99.8)	96.4 (89.8–99.2)	91.1 (87.6–93.8)
>12	860 (97.7)	20 (2.3) **^,c^	2 (5.3)	36 (94.7)	64.3 (50.4–76.6)	99.8 (99.2–100)	94.7 (82.3–99.4)	97.7 (96.5–98.6)
**the 3rd trimester of pregnancy**								
All the women	935 (95.4)	45 (4.6)	4 (4.0)	95 (96)	67.9 (59.4–75.5)	99.6 (98.9–99.9)	96.0 (90.0–98.9)	95.4 (93.9–96.6)
Maternal age (years)								
<30	530 (94.3)	32 (5.7) *	4 (6.2)	61 (93.8)	65.6 (55.0–75.1)	99.3 (98.1–99.8)	93.8 (85–98.3)	94.3 (92.1–96.1)
≥30	399 (96.8)	13 (3.2)	0 (0)	33 (100)	71.7 (56.5–84.0)	100 (98.6–100)	100 (84.7–100)	96.8 (94.7–98.3)
Parity								
0	482 (95.3)	24 (4.7)	3 (5.9)	48 (94.1)	66.7 (54.6–77.3)	99.4 (98.2–99.9)	94.1 (83.8–98.8)	95.3 (93.0–96.9)
1	326 (95.9)	14 (4.1)	1 (3.4)	28 (96.6)	66.7 (50.5–80.4)	99.7 (98.3–100)	96.6 (82.2–99.9)	95.9 (93.2–97.7)
≥2	127 (94.8)	7 (5.2)	0 (0)	19 (100)	73.1 (52.2–88.4)	100 (95.7–100)	100 (75.1–100)	94.8 (89.5–97.9)
Marital status								
Married	773 (96.5)	28 (3.5) **	2 (4.4)	43 (95.6)	60.6 (48.3–72.0)	99.7 (99.1–100.0)	95.6 (84.9–99.5)	96.5 (95.0–97.7)
Single	161 (90.4)	17 (9.6)	2 (3.7)	52 (96.3)	75.4 (63.5–84.9)	98.8 (95.6–99.9)	96.3 (87.3–99.5)	90.4 (85.1–94.3)
Maternal education								
≤9	20 (87)	3 (13)	0 (0)	31 (100)	91.2 (76.3–98.1)	100 (76.2–100)	100 (83.8–100)	87 (66.4–97.2)
10–12	265 (91.7)	24 (8.3) **^,b^	2 (3.8)	50 (96.2)	67.6 (55.7–78.0)	99.3 (97.3–99.9)	96.2 (86.8–99.5)	91.7 (87.9–94.6)
>12	649 (97.3)	18 (2.7) *^,c^	2 (12.5)	14 (87.5) *^,c^	43.8 (26.4–62.3)	99.7 (98.9–100)	87.5 (61.7–98.4)	97.3 (95.8–98.4)

^a^ ≤9, 10–12; ^b^ 10–12, >12; ^c^ ≤9, >12; * *p* < 0.05; ** *p* < 0.001; the numbers might not sum up to the total sample size as some missing data were observed (described in the Materials and Methods section of the manuscript).

**Table 5:** Sensitivity (Se), specificity (Sp), positive and negative predictive values (PV+, PV−) according to the saliva cotinine cut-off of 1.5 ng/mL.

Characteristics	Not Exposed to Passive Smoking ° *n* (%)		Exposed to Passive Smoking ° *n* (%)		Se (95%CI)	Sp (95% CI)	PV+ (95% CI)	PV− (95% CI)
	<1.5 ng/mL	≥1.5 ng/mL	<1.5 ng/mL	≥1.5 ng/mL				
**the 1st trimester of pregnancy**								
All the women	744 (70.7)	309 (29.3)	77 (36.8)	132 (63.2)	29.9 (25.7–34.4)	90.6 (88.4–92.5)	63.2 (56.2–69.7)	70.7 (67.8–73.4)
Maternal age (years)								
<30	373 (67.6)	179 (32.4) *	58 (37.7)	96 (62.3)	34.9 (29.3–40.9)	86.5 (83–89.6)	62.3 (54.2–70.0)	67.6 (63.5–71.5)
≥30	362 (74.0)	127 (26.0)	19 (34.5)	36 (65.5)	22.1 (16.0–29.2)	95 (92.3–97.0)	65.5 (51.4–77.8)	74 (69.9–77.9)
Parity								
0	370 (69.9)	159 (30.1)	50 (37.9)	82 (62.1)	34 (28.1–40.4)	88.1 (84.6–91)	62.1 (53.3–70.4)	69.9 (65.8–73.8)
1	262 (70.1)	112 (29.9)	19 (39.6)	29 (60.4)	20.6 (14.2–28.2)	93.2 (89.6–95.9)	60.4 (45.3–74.2)	70.1 (65.1–74.7)
≥2	112 (74.7)	38 (25.3)	8 (27.6)	21 (72.4)	35.6 (23.6–49.1)	93.3 (87.3–97.1)	72.4 (52.8–87.3)	74.7 (66.9–81.4)
Marital status								
Married	646 (72.6)	244 (27.4) **	57 (43.8)	73 (56.2) *	23 (18.5–28.1)	91.9 (89.6–93.8)	56.2 (47.2–64.8)	72.6 (69.5–75.5)
Single	98 (60.1)	65 (39.9)	20 (25.3)	59 (74.7)	47.6 (38.5–56.7)	83.1 (75.0–89.3)	74.7 (63.6–83.8)	60.1 (52.2–67.7)
Maternal education								
≤9	3 (23.1)	10 (76.9) **^,a^	6 (27.3)	16 (72.7)	61.5 (40.6–79.8)	33.3 (7.5–70.1)	72.7 (49.8–89.3)	23.1 (5.0–53.8)
10–12	167 (67.3)	81 (32.7)	33 (31.4)	72 (68.6) *^,b^	47.1 (38.9–55.3)	83.5 (77.6–88.4)	68.6 (58.8–77.3)	67.3 (61.1–73.1)
>12	574 (72.6)	217 (27.4) **^,c^	38 (46.3)	44 (53.7)	16.9 (12.5–22)	93.8 (91.6–95.6)	53.7 (42.3–64.7)	72.6 (69.3–75.6)
**the 3rd trimester of pregnancy**								
All the women	590 (71.4)	236 (28.6)	51 (37.5)	85 (62.5)	26.5 (21.7–31.7)	92 (89.7–94.0)	62.5 (53.8–70.6)	71.4 (68.2–74.5)
Maternal age (years)								
<30	316 (69.8)	137 (30.2)	38 (37.6)	63 (62.4)	31.5 (25.1–38.4)	89.3 (85.6–92.3)	62.4 (52.2–71.8)	69.8 (65.3–74.0)
≥30	268 (73.0)	99 (27.0)	13 (37.1)	22 (62.9)	18.2 (11.8–26.2)	95.4 (92.2–97.5)	62.9 (44.9–78.5)	73 (68.2–77.5)
Parity								
0	294 (73.0)	109 (27.0)	33 (35.9)	59 (64.1)	35.1 (27.9–42.8)	89.9 (86.1–93.0)	64.1 (53.5–73.9)	73 (68.3–77.2)
1	213 (68.7)	97 (31.3)	11 (42.3)	15 (57.7)	13.4 (7.7–21.1)	95.1 (91.4–97.5)	57.7 (36.9–76.6)	68.7 (63.2–73.8)
≥2	83 (73.5)	30 (26.5)	7 (38.9)	11 (61.1)	26.8 (14.2–42.9)	92.2 (84.6–96.8)	61.1 (35.7–82.7)	73.5 (64.3–81.3)
Marital status								
Married	503 (71.7)	199 (28.3)	33 (39.8)	50 (60.2)	20.1 (15.3–25.6)	93.8 (91.5–95.7)	60.2 (48.9–70.8)	71.7 (68.2–75.0)
Single	86 (69.9)	37 (30.1)	18 (34.0)	35 (66.0)	48.6 (36.7–60.7)	82.7 (74.0–89.4)	66 (51.7–78.5)	69.9 (61.0–77.9)
Maternal education								
≤9	5 (41.7)	7 (58.3)	2 (18.2)	9 (81.8)	56.3 (29.9–80.2)	71.4 (29.0–96.3)	81.8 (48.2–97.7)	41.7 (15.2–72.3)
10–12	134 (63.8)	76 (36.2) *^,b^	29 (39.2)	45 (60.8)	37.2 (28.6–46.4)	82.2 (75.5–87.7)	60.8 (48.8–72.0)	63.8 (56.9–70.3)
>12	450 (74.6)	153 (25.4) *^,c^	20 (39.2)	31 (60.8)	16.8 (11.7–23.1)	95.7 (93.5–97.4)	60.8 (46.1–74.2)	74.6 (71.0–78.1)

° Restricted to non-smokers; self-reported passive smoking is acceptance of smoking at home; ^a^ ≤9, 10–12; ^b^ 10–12, >12; ^c^ ≤9, >12; * *p* < 0.05; ** *p* < 0.001; the numbers might not sum up to the total sample size as some missing data were observed (described in the Materials and Methods section of the manuscript).

## Supplementary Materials

The following are available online at www.mdpi.com/1660-4601/13/12/1216/s1, Table S1: Active smoking during pregnancy (1st, 2nd and 3rd trimester) according to maternal characteristics, Table S2: Passive smoking during pregnancy (1st trimester) according to maternal characteristics, Table S3: Passive smoking during pregnancy (2nd trimester) according to maternal characteristics, Table S4: Passive smoking during pregnancy (3rd trimester) according to maternal characteristics, Figure S1: Histograms of log (cotinine) in each trimester of pregnancy and self-reported active and passive smoking status.
