# Supplementary material for: Estimation of Saliva Cotinine Cut-Off Points for Active and Passive Smoking during Pregnancy—Polish Mother and Child Cohort (REPRO_PL)
Source: Int J Environ Res Public Health. 2016 Dec 8;13(12):1216. doi: 10.3390/ijerph13121216 (PMC5201357; doi:10.3390/ijerph13121216)
Supplement: Supplementary file 1 [file ijerph-13-01216-s001.pdf]

Supplementary Materials: Estimation of Saliva Cotinine Cut-Off Points for Active and Passive Smoking during Pregnancy—Polish Mother and Child Cohort (REPRO_PL)

Kinga Polanska, Anna Krol, Pawel Kaluzny, Danuta Ligocka, Karolina Mikolajewska, Seif Shaheen, Robert Walton and Wojciech Hanke

Table S1. Active smoking during pregnancy (1st, 2nd and 3rd trimester) according to maternal characteristics.

Characteristics *n* (%)	1st Trimester			2nd Trimester			3rd Trimester		
	Missing	Non-Smokers *n* (%)	Smokers *n* (%)	Missing	Non-Smokers *n* (%)	Smokers *n* (%)	Missing	Non-Smokers *n* (%)	Smokers *n* (%)
All women; *n* = 1771	70	1505 (88.5%)	196 (11.5%)	273	1356 (90.5%)	142 (9.5%)	376	1273 (91.3%)	122 (8.7%)
Maternal age (years) [a]									
<30; *n* = 963 (57.0)	4	826 (86.1%)	133 (13.9%) **	106	761 (88.8%)	96 (11.2%) *	155	729 (90.2%)	79 (9.8%)
≥30; *n* = 725 (43.0)	5	659 (91.5%)	61 (8.5%)	100	580 (92.8%)	45 (7.2%)	153	530 (92.7%)	42 (7.3%)
Parity [a]									
0; *n* = 874 (51.1)	6	772 (88.9%)	96 (11.1%)	107	696 (90.7%)	71 (9.3%)	153	659 (91.4%)	62 (8.6%)
≥1; *n* = 836 (48.9)	3	733 (88.0%)	100 (12.0%)	105	660 (90.3%)	71 (9.7%)	162	614 (91.1%)	60 (8.9%)
Marital status [a]									
Single; *n* = 384 (22.5)	1	293 (76.5%)	90 (23.5%) **	53	256 (77.3%)	75 (22.7%) **	72	244 (78.2%)	68 (21.8%) **
Married; *n* = 1322 (77.5)	5	1211 (92.0%)	106 (8.0%)	156	1099 (94.3%)	67 (5.7%)	240	1028 (95.0%)	54 (5.0%)
Maternal education [a]									
≤9; *n* = 80 (4.7)	0	39 (48.8%)	41 (51.3%) [b], **	9	34 (47.9%)	37 (52.1%) [b], **	14	32 (48.5%)	34 (51.5%) [b], **
10–12; *n* = 527 (30.9)	3	421 (80.3%)	103 (19.7%) [c], **	57	392 (83.4%)	78 (16.6%) [c], **	90	374 (85.6%)	63 (14.4%) [c], **
>12; *n* = 1097 (64.4)	2	1044 (95.3%)	51 (4.7%) [d], **	142	928 (97.2%)	27 (2.8%) [d], **	206	866 (97.2%)	25 (2.8%) [d], **

[a] Missing maternal age data *n* = 83, missing parity data *n* = 61, missing marital status data *n* = 65, missing educational data *n* = 67; [b] ≤9 vs. 10–12; [c] 10–12 vs. >12; [d] ≤9 vs. >12; * $p < 0.05$; ** $p < 0.001$; the numbers might not sum up to the total sample size as some missing data were observed (described in the methods section of the manuscript).

Table S2. Passive smoking during pregnancy (1st trimester) according to maternal characteristics.

Characteristics	Husband/Partner Smoking			ETS			Smoking Allowed at Home		
	Missing	No	Yes	Missing	No	Yes	Missing	No	Yes
		n (%)	*n* (%)		*n* (%)	*n* (%)		*n* (%)	*n* (%)
All women (*n* = 1771)	92	1108 (66.0%)	571 (34.0%)	71	912 (53.6%)	788 (46.4%)	80	1308 (77.4%)	383 (22.6%)
Maternal age (years) [a]									
<30 (*n* = 963)	18	570 (60.3%)	375 (39.7%) **	5	453 (47.3%)	505 (52.7%) **	8	683 (71.5%)	272 (28.5%) **
≥30 (*n* = 725)	13	525 (73.7%)	187 (26.3%)	5	445 (61.8%)	275 (38.2%)	11	605 (84.7%)	109 (15.3%)
Parity [a]									
0 (*n* = 874)	17	561 (65.5%)	296 (34.5%)	4	448 (51.5%)	422 (48.5%)	11	641 (74.3%)	222 (25.7%) *
≥1 (*n* = 836)	14	547 (66.5)	275 (33.5%)	6	464 (55.9%)	366 (44.1)	8	667 (80.6%)	161 (19.4%)
Marital status [a]									
Single (*n* = 384)	9	178 (47.5%)	197 (52.5%) **	1	132 (34.5%)	251 (65.5%) **	4	218 (57.4%)	162 (42.6%) **
Married (*n* = 1322)	19	929 (71.3%)	374 (28.7%)	6	780 (59.3%)	536 (40.7%)	11	1090 (83.1%)	221 (16.9%)
Maternal education [a]									
≤9 (*n* = 80)	0	25 (31.3%)	55 (68.8%) [b,] **	1	14 (17.7%)	65 (82.3%) [b,] **	0	20 (25.0%)	60 (75.0%) [b,] **
10–12 (*n* = 527)	7	264 (50.8%)	256 (49.2%) [c,] **	2	199 (37.9%)	326 (62.1%) [c,] **	6	328 (63.0%)	193 (37.0%) [c,] **
>12 (*n* = 1097)	20	818 (76.0%)	259 (24.0%) [d,] **	3	698 (63.8%)	396 (36.2%) [d,] **	10	959 (88.2%)	128 (11.8%) [d,] **

[a] Missing maternal age data *n* = 83, missing parity data *n* = 61, missing marital status data *n* = 65, missing educational data *n* = 67; [b] ≤9 vs. 10–12; [c] 10–12 vs. >12; [d] ≤9 vs. >12; * $p < 0.05$; ** $p < 0.001$; the numbers might not sum up to the total sample size as some missing data were observed (described in the methods section of the manuscript); ETS: environmental tobacco smoke.

Table S3. Passive smoking during pregnancy (2nd trimester) according to maternal characteristics.

Characteristics	Husband/Partner Smoking			ETS			Smoking Allowed at Home		
	Missing	No	Yes	Missing	No	Yes	Missing	No	Yes
		n (%)	*n* (%)		*n* (%)	*n* (%)		*n* (%)	*n* (%)
All women (*n* = 1771)	290	1033 (69.8%)	448 (30.2%)	290	1139 (76.9%)	342 (23.1%)	287	1193 (80.4%)	291 (19.6%)
Maternal age (years) [a]									
<30 (*n* = 963)	114	552 (65.0%)	297 (35.0%) **	114	603 (71.0%)	246 (29.0%) *	110	644 (75.5%)	209 (24.5%) *
≥30 (*n* = 725)	109	470 (76.3%)	146 (23.7%)	109	522 (84.7%)	94 (15.3%)	109	534 (86.7%)	82 (13.3%)
Parity [a]									
0 (*n* = 874)	114	523 (68.8%)	237 (31.2%)	115	568 (74.8%)	191 (25.2%)	111	588 (77.1%)	175 (22.9%) *
≥1 (*n* = 836)	115	510 (70.7%)	211 (29.3%)	114	571 (79.1%)	151 (20.9%)	115	605 (83.9%)	116 ((16.1%)
Marital status [a]									
Single (*n* = 384)	57	176 (53.8%)	151 (46.2%) *	56	177 (54.0%)	151 (46.0%) *	55	201 (61.1%)	128 (38.9%) *
Married (*n* = 1322)	169	856 (74.2%)	297 (25.8%)	170	961 (83.4%)	191 (16.6%)	168	991 (85.9%)	163 (14.1%)
Maternal education [a]									
≤9 (*n* = 80)	8	24 (33.3%)	48 (66.7%) [b,*]	8	22 (30.6%)	50 (69.4%) [b,*]	8	30 (41.7%)	42 (58.3%) [b,*]
10–12 (*n* = 527)	62	270 (58.1%)	195 (41.9%) [c,*]	62	294 (63.2%)	171 (36.8%) [c,*]	62	305 (65.6%)	160 (34.4%) [c,*]
>12 (*n* = 1097)	154	739 (78.4%)	204 (21.6%) [d,*]	154	822 (87.2%)	121 (12.8%)	151	857 (90.6%)	89 (9.4%) [d,*]

[a] Missing maternal age data *n* = 83, missing parity data *n* = 61, missing marital status data *n* = 65, missing educational data *n* = 67, [b] ≤9 vs. 10–12; [c] 10–12 vs. >12; [d] ≤9 vs. >12; * $p < 0.001$; the numbers might not sum up to the total sample size as some missing data were observed (described in the methods section of the manuscript).

Table S4. Passive smoking during pregnancy (3rd trimester) according to maternal characteristics.

Characteristics	Husband/Partner Smoking			ETS			Smoking Allowed at Home		
	Missing	No	Yes	Missing	No	Yes	Missing	No	Yes
		n (%)	*n* (%)		*n* (%)	*n* (%)		*n* (%)	*n* (%)
All women (*n* = 1771)	396	959 (69.7%)	416 (30.3%)	398	1080 (78.7%)	293 (21.3%)	397	1105 (80.4%)	269 (19.6%)
Maternal age (years) [a]									
<30 (*n* = 963)	165	523 (65.5%)	275 (34.5%) **	166	585 (73.4%)	212 (26.6%) **	165	606 (75.9%)	192 (24.1%) *
≥30 (*n* = 725)	163	426 (75.8%)	136 (24.2%)	164	484 (86.3%)	77 (13.7%)	164	486 (86.6%)	75 (13.4%)
Parity [a]									
0 (*n* = 874)	166	491 (69.4%)	217 (30.6%)	166	534 (75.4%)	174 (24.6%) *	165	547 (77.2%)	162 (22.8%) *
≥1 (*n* = 836)	169	468 (70.2%)	199 (29.8%)	171	546 (82.1%)	119 (17.9%)	171	558 (83.9%)	107 (16.1%)
Marital status [a]									
Single (*n* = 384)	77	164 (53.4%)	143 (46.6%) **	75	172 (55.7%)	137 (44.3%) **	76	181 (58.8%)	127 (41.2%) **
Married (*n* = 1322)	255	794 (74.4%)	273 (25.6%)	259	907 (85.3%)	156 (14.7%)	257	923 (86.7%)	142 (13.3%)
Maternal education [a]									
≤9 (*n* = 80)	14	21 (31.8%)	45 (68.2%) [b,] **	14	20 (30.3%)	46 (69.7%) [b,] **	14	21 (31.8%)	45 (68.2%) [b,] **
10–12 (*n* = 527)	97	244 (56.7%)	186 (43.3%) [c,] **	95	286 (66.2%)	146 (33.8%) [c,] **	97	290 (67.4%)	140 (32.6%) [c,] **
>12 (*n* = 1097)	219	694 (79.0%)	184 (21.0%) [d,] **	223	773 (88.4%)	101 (11.6%) [d,] **	220	793 (90.4%)	84 (9.6%) [d,] **

[a] Missing maternal age data *n* = 83; missing parity data *n* = 61; missing marital status data *n* = 65; missing educational data *n* = 67; [b] ≤9 vs. 10–12; [c] 10–12 vs. >12; [d] ≤9 vs. >12; * $p < 0.05$; ** $p < 0.001$; the numbers might not sum up to the total sample size as some missing data were observed (described in the methods section of the manuscript).

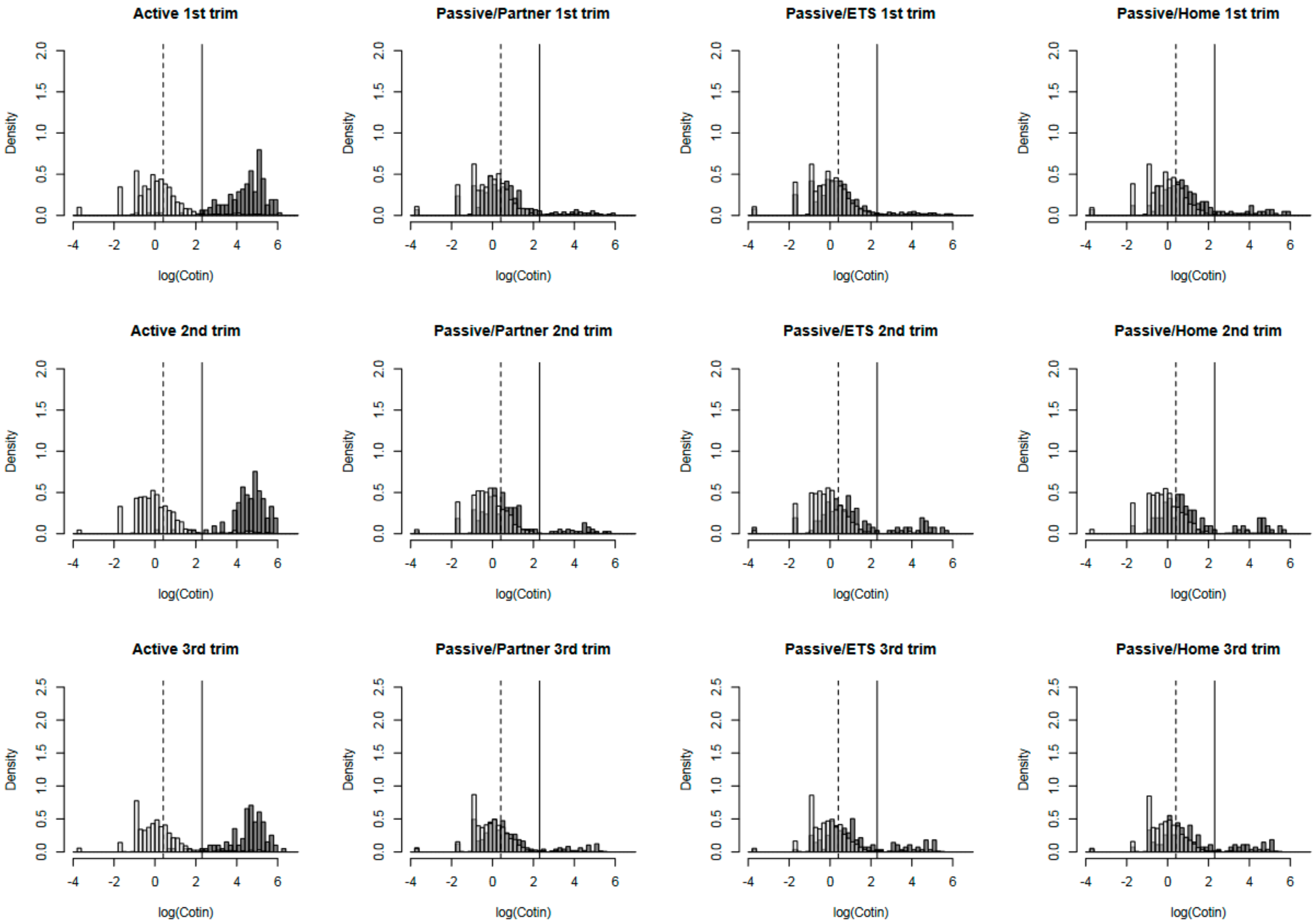

Figure S1. Histograms of log (cotinine) in each trimester of pregnancy and self-reported active and passive smoking status. Non-exposed—light gray; Exposed—dark gray; Overlapping parts of histograms—moderate gray; Vertical lines indicate cut-off points for cotinine levels: the continuous vertical line corresponds to 10 ng/mL as the cut-off point for active smoking, the dotted vertical line corresponds to 1.5 ng/mL as the cut-off point for passive smoking. ETS: environmental tobacco smoke.
